# Participatory management and sustainable ecosystem management indicators

**DOI:** 10.1038/s41598-024-83677-w

**Published:** 2024-12-30

**Authors:** Saeed Maghsoodi, Seyedeh Khadijeh Mahdavi, Mohammadreza Shahraki, Mohsen Sharafatmandrad

**Affiliations:** 1 Department of Natural Resource, Nour Branch, Islamic Azad University, Nour, Iran; 2 Researcher of Rural Development and Social Issues in the Field of Natural Resources and Agriculture, Gorgan, Iran; 3 Department of Ecological Engineering, Faculty of Natural Resources, University of Jiroft, 8th km of Jiroft - Bandar Abbas road, P.O. Box: 7867161167, Jiroft, Iran

**Keywords:** Livelihood, Participation, Pastoralism, Rangeland, Social capital, Socioeconomic scenarios, Sustainability, Environmental social sciences

## Abstract

The United Nations has implemented projects focusing on the participation of local communities in economic, social and ecological criteria in different countries. The present study aimed to assess the effects of such projects on sustainable ecosystem management indicators in the Tilabad watershed, Golestan province, Iran. The statistical population includes 99 pastoralists from 3 pastoral units, of which 80 pastoralists were selected as the sample size and sampled by stratified random sampling technique. The measurement tool was a researcher-made questionnaire. The results showed that 41.3% of the respondents (the highest frequency) evaluated the effectiveness of project as good. The implementation of the participatory project has had a positive and significant effect on the sustainable ecosystem management (β = 0.703 and t = 12.756). The greatest effectiveness is related to the social (β = 0.671 and t = 12.146) and economic (β = 0.644 and t = 8.615) criteria. Age, experience of animal husbandry (*P* ≤ 0.05), the number of animals, and the amount of income from animal husbandry (*P* ≤ 0.01) had a positive and significant relationship with the project effectiveness on sustainable ecosystem management in the area. It can be concluded that participatory approaches can lead to sustainable ecosystem management.

## Introduction

Local participation in the natural resource planning and management is one of the key components in social-ecological systems of natural resource governance that may lead to economic and social development^1,2,3,4^. In fact, natural resources protection requires integrated management, which cannot happen without the participation of local communities^5,6,7^. Natural resource management and biodiversity protection strategies are shifted from focusing on centralized planning and management of government organizations to participatory strategies with balanced social, economic and environmental goals^8^. The participation of local communities in the conservation and restoration of natural resources is considered as a necessity. People’s participation in decision-making and implementation of restoration and conservation activities is the most effective strategy for integrating sustainability in natural resources^9,10^. The sustainability of natural resources is achieved when the exploitation is based on all the factors affecting the sustainability of the ecosystem^11^. The natural resources sustainability is directly related to the economic, social and environmental sustainability^12^. The urgent need of locals for livelihood provisioning is one of the important problems governing most pastoral units^11^. Therefore, the stability of social, economic, cultural, and environmental conditions is the basis for the sustainability of natural resources, doubling the importance of sustainable development^13^. In other words, the sustainable management of natural resources is directly related to the socio-economic structure of locals and the ecological status of the ecosystems^14^. Therefore, the sustainable natural resources management intends to interrelate local communities and government institutions through a systemic view by combining ecological, socio-cultural, and political principles^15^. Based on the Wallerstein & Duran^16^, community-based participatory approach are defined as “approaches that provide a framework to equitably involve community members, researchers and other stakeholders in the research process, recognizing and maximizing the importance of their diverse contributions”.

Community-based participatory approaches can affect community governance and create sustainable social capital by changing the infrastructure and economic efficiency of local communities (9). In fact, participation changes social relations and capitals, which is an important structure for interpreting the behaviors of local stakeholders^17^. In addition, participation significantly affects intra-group trust and trust in the government^1, 18^. In this regard, community-based management of natural resources has had a significant impact on conservatory behaviors of locals by variables such as information level, leadership, knowledge exchange, and laws^19^. In addition, local knowledge-based methods have enabled marginal local communities to protect natural resources, improving their food security, income, and living conditions^20^. Increasing the amount of income, improving social and economic infrastructure through the participation and cooperation are signs of the empowerment of the local communities^21^. Therefore, the increase in indigenous communities’ capacity leads to the improved local participation, increased awareness and income level, improved livelihood, and improved marketing of products produced in villages^22^.

Many projects in the field of natural resource management with the participation of local communities have been implemented or are currently being implemented worldwide^19^. For example, the international project of participatory management of natural resources and rural development was carried out with the financial support of UNDP and GEF^23^. This project aimed to restore the degraded areas and also improve the living conditions of local communities, taking into account the main aspects of sustainable development (social, economic and environmental) by using participatory methods and empowering local communities^23, 24^. In this regard, a project was implemented in the Tilabad watershed, Golestan province, Iran in 2013. The most important goals of the project were^1^ to remove the key obstacles to sustainable land management^2^, to restore biodiversity, (3) to increase the capacity of degraded lands and rangeland landscapes^4^, to exploit the services and products obtained from the ecosystem^5^, to create sustainable livelihood, food security^6^, to combat desertification through promoting integrated participatory activities, and (7) to increase national and local capacity^24^. After more than a decade of the project implementation, the rangelands were restored by taking advantage of the local communities’ participation. Effective measures have been taken to empower local communities by creating job opportunities, i.e. the cultivation and processing of medicinal plants, especially saffron, promoting the production of handicrafts, and expanding ecotourism. Producing forest saplings and rangeland shrubs and their planting with the participation of locals were other activities that have created seasonal and permanent sources of income for the local communities in the region. Now, the question that comes to mind is that whether the implementation of programs and policies of participatory management of natural resources and rural development has been effective on the economic, social and environmental indicators of sustainable management? The answer can bring policies and management plans into a more stable environment in the future. Therefore, this study aimed to^1^ assess the performance of the natural resources participatory management and rural development project^2^, assess the relationship between personal characteristics of pastoralists and sustainable management of natural resources participatory management and rural development, and (3) assess the effect of participatory management project on the sustainable natural resources management.

## Materials and methods

### Study area

**Fig. 1:**
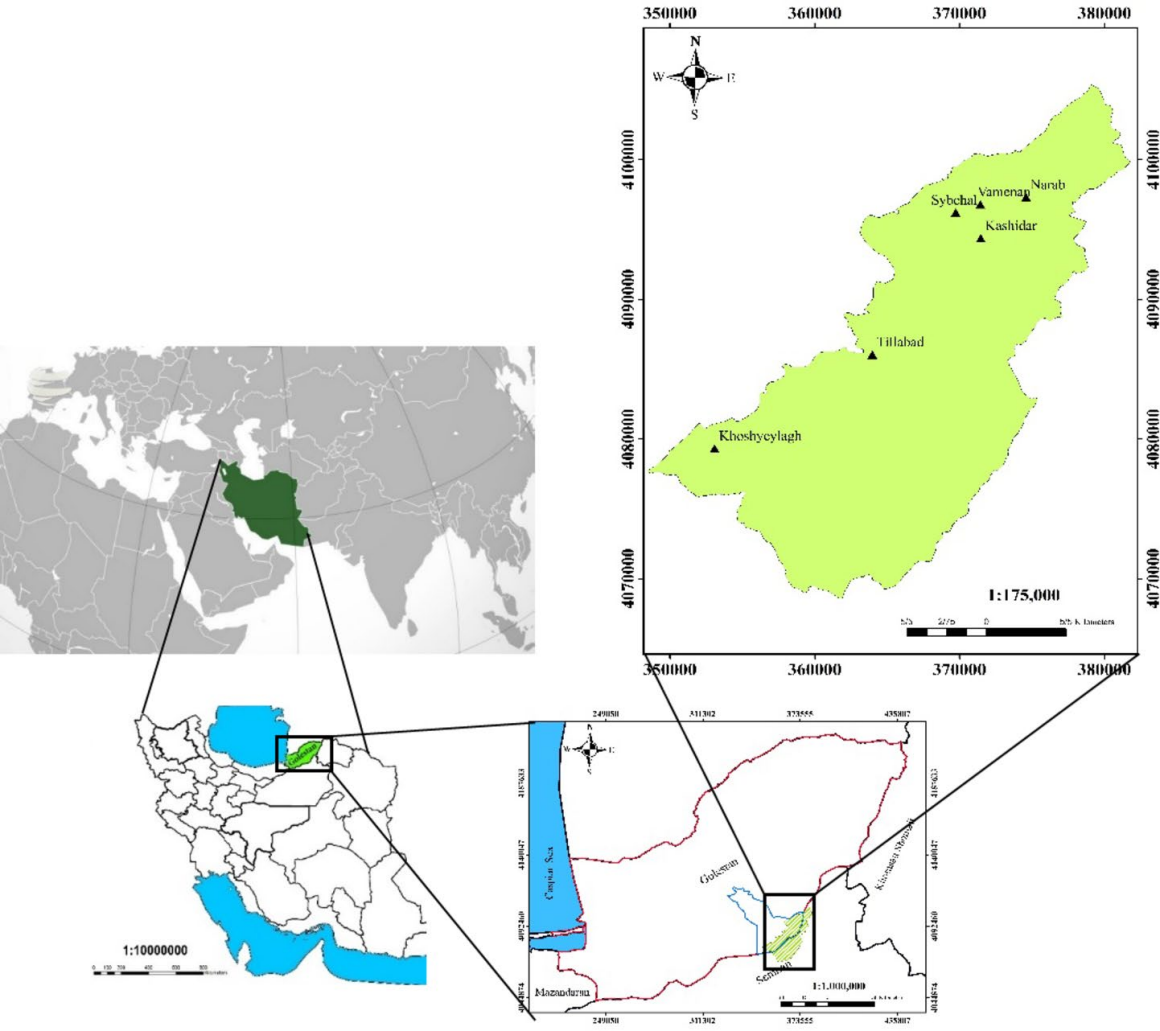
The map of Tilabad watershed and its villages. Source: Mapped by the authors using ArcGIS Desktop V. 10.8 (http://www.arcgis.com).

Tilabad watershed with an area of 48329 hectares is located in Golestan province, Iran (55°11’42” to 55°40’15” E and 36°45’12” to 37°07’31"N). The average annual rainfall is 343.19 mm and the average annual temperature is 10.7° Celsius. According to de Martonne classification, the climate of the region is semi-arid. The main land covers in the region are rangelands (28452 hectares) and forests (5210 hectares). There are several important villages in the region, including Kashidar, Vamanan, Sibchal, Narab, Golestan, Talobin, Tilabad, and Khosh Yeylagh (Fig. 1). The main occupation of the residents is traditional animal husbandry (pastoralism) and agriculture. The pastoralists in the region belonge to three pastoral units (i.e. Khosh Yeylagh, Tilabad, and Kashidar). The pastoralists have a license to graze their herd in the rangelands for about 4 months from mid-spring to the end of summer.

### Methods

A combination of quantitative and qualitative methods was used in this study, which is a survey research. In the qualitative section, interviews were conducted with local experts to identify the research criteria and indices. In the quantitative section, obtained data were statistically analyzed.

### Statistical population and sampling

The statistical population included 99 pastoralists from 3 pastoral units (Khosh Yeylagh with 22 pastoralists, Tilabad with 17 pastoralists, and Kashidar with 52 pastoralists), of which 80 pastoralists were selected as the sample size using Krejcie & Morgan Table^25^. Pastoralists were randomly selected by stratified random sampling technique based on the proportion of pastoralist in each pastoral unit to the sample size.

### Data collection

A researcher-made questionnaire was used to collect data. The questionnaire was compiled in three parts according to the main research objectives. The first part was related to the personal characteristics of the respondents (Table 1).

**Table 1:** Personal characteristics of the respondents.

Criteria	Indices	Defination	Scale
Personal characteristics	Marital status	Married Single	Nominal
	Owning agricultural land	Yes No	
	Having a job other than animal husbandry	Yes No	
	Membership in rural organizations and cooperatives	Yes No	
	Number of livestock	The number of small livestock (sheep and goats) owned	Interval
	Number of household members	The number of people who are covered by the head of the family	
	Age	The years a person has lived.	Ratio
	Animal husbandry background	The years of experience a person has in. animal husbandry	
	Distance from residence to rangeland	The nearest distance between a pastoralist’s residence and rangeland	
	Education	The level of literacy based on the academic degree.	Ordinal
	Annual income from. animal husbandry	Pastoralist’s income for livestock activities during one year	

The second part of the questionnaire was related to the performance of the natural resources participatory management and rural development project. This part was evaluated using a five-point Likert scale with 10 indices (Table 2).

**Table 2:** The indices used to assess the performance of the natural resources participatory management and rural development project.

Criteria	Indices	Scale
The performance of the natural resources participatory management and rural development project	Providing the context for the participation of local communities in the restoration of rangelands	Ordinal
	Creation of new job opportunities	
	Diversification of income sources in rural households	
	Participation of rural organizations in project implementation	
	Raising the level of knowledge and awareness of the locals to meet the project goals	
	Actualizing and promoting recreational potentials	
	Improving infrastructure and construction services in villages	
	Reducing the dependence of local communities on natural resources	
	Increasing rangeland restoration practices	

In the third part, sustainable rangeland management was assessed in four criteria with 82 indices (Table 3). The indices were evaluated by a five-point Likert scale (very little with a numerical value of 1, low with a numerical value of 2, medium with a numerical value of 3, high with a numerical value of 4, and very much with a numerical value of 5). Composite reliability coefficient (CR) and Cronbach’s alpha coefficient were used to determine the reliability of the measurement tool^26^. The calculated Cronbach’s alpha coefficient value was more than 0.7, showing appropriate reliability of the research measurement tool.

**Table 3:** The criteria, indicators, and indices used to assess the sustainable rangeland management.

Criteria	Indicators	Indices
Social	Social trust	Trust in governmental organization; Trust in local councils Trust in the villagers Trust in other pastoralists
	Social participation	Participation in rangeland restoration Participating in rangeland conservation and exclosure Cooperation in the restoration of water fountain such as springs Participation in the proper distribution of abreuvoirs in the rangeland Participation in educational and extension courses Participation in regional ceremonies and meetings Observing the time of entry and exit of livestock to the rangeland The use of agricultural tools for rangeland restoration Compliance with grazing capacity Participation in collective decision-making in the village
	Social awareness	Changing the attitude towards group activities in the rangeland Membership in rural cooperatives and small funds More referrals to extension experts, facilitators and consultants for booming animal husbandry Increasing the awareness of pastoralists in different ways Increasing knowledge and information related to alternative income and livelihood sources Informing pastoralists about the rules and encouraging them to comply
	Social cohesion and solidarity	Solving problems and disputes by elders Strengthening relationships and interactions Helping each other in times of trouble The existence of a spirit of solidarity and cooperation Increasing place attachment and improving livability Reducing the number of violations in the rangeland
	Social interactions and communication	Raising the social status of pastoralists in rangeland management Relationships Outside the pastoral units Promotion of emotional relationships between pastoralists More interaction of pastoralists within the pastoral units
Economic	Development of income sources and livelihood	Changing income and livelihood sources Increasing income from the sale of livestock products Development of income sources through the processing of dairy and meat products
	Innovation in job creation and its quality	Access to jobs other than animal husbandry in the village Increasing job opportunities in agricultural and horticultural affairs such as medicinal plants Increasing job opportunities related to handicrafts Increasing job and income opportunities related to tourism and eco-tourism and providing services Increasing home occupation opportunities (processing and packaging of products) Job opportunities and creating employment in rangeland section Acceptance of pastoralists membership in small rural funds Implementation of livestock and rangeland insurance
	Financial support and investment	Increasing investment in rural businesses and preventing migration Increasing the provision of loans and facilities to pastoralists Investment of pastoralists in rangeland restoration
	Strengthening production and creating a market	Increasing production in concentrated animal husbandry and beekeeping Strengthening production and creating a market Access to local and regional markets and distribution of local products Quantitative and qualitative improvement of rural production to increase income Development of non-forage products in rangelands
Environmental	Conservation	Rangeland exclosure and protection of critical areas for natural reproduction Reduction of fires in rangelands Distribution of fossil fuel to reduce consumption of fuel plants The use of solar panels to reduce consumption of fuel plants Preventing the entry of non-native herdsmen Biological and mechanical control of pests and plant diseases
	Restoration	Restoration of sloping rangelands through interseeding Increasing the planting area in the rangeland Cultivation of fruit trees and forage plants in sloping lands Increasing the seeding area in the rangeland
		Fertilizing in rangelands to strengthen vegetation Strengthening springs and creating new streams Distribution of abreuvoirs in rangelands Restoration of fountains in the rangeland Bee breeding in the rangeland Compliance with the grazing capacity to reduce the grazing pressure on the rangeland Aggregation of small pastoralists
Service-construction	Educational	Using indigenous knowledge in rangeland management Providing extension and educational courses on the importance of rangelands and their services Training of pastoralists about the proper exploitation and protection of rangelands Increasing the training courses to empower pastoralists; Acquainting farmers with alternative jobs Increasing educational facilities for the family in the village
	Sanitary	Improving household health facilities Increasing veterinary services Increasing the fight against pests and livestock diseases Improving the health status of drinking water
	Construction- infrastructural	Distribution of fossil fuel to reduce consumption of fuel plants Providing subsidized forage to reduce entry to the rangeland Provision of access routes to the rangelands for the traffic of pastoralists Development and contextualization of eco-tourism Establishing small funds for rural pastoralists Improving amenities such as water, electricity, etc. in the village.

### Data analyses

Descriptive statistics was used to show the main features of personal characteristics. The mean and standard deviation were used to assess the performance of the natural resources participatory management and rural development project. The interval of standard deviation from the mean (ISDM) was calculated to classify the performance of the natural resources participatory management and rural development project. Based on the sum of the values, strategies were classified into four classes:

Weak: ISDM < Mean-St.d.

Medium: Mean-St.d ≤ ISDM < Mean.

Good: Mean ≤ ISDM < Mean + St.d.

Excellent: Mean + St.d ≤ ISDM.

Spearman’s correlation coefficient was used to investigate the relationship between the personal characteristics of pastoralists and the sustainable management of the natural resources participatory management and rural development indicators.

Structural equation modeling (known as the partial least squares approach or variance-based approach) was used to assess the effect of participatory management project on the sustainable natural resources management criteria. Hypothetical patterns of direct and indirect relationships between a set of observed and latent variables are investigated in structural equation modeling. Latent variables are the main factors that are displayed in a model or conceptual model. Observed variables are items or questions related to measuring the main factors. This method is a special causal structure between a set of latent variables and observable variables. Using the structural equation modeling, the relationships between the latent variables and the relationships between measurement items of each latent variable and the related variable were investigated. The factor load of each index was first calculated to investigate the relationship between the latent and observable variables. Factor load is confirmed for all indices if its value is equal or greater than 0.5. The path coefficient was used to show the e causal relationship between the latent and observable variables. The strength of the relationship between the latent and observable variables is represented by the factor loading. SmartPLS 3 was used for structural equation modeling. In the partial least squares (PLS) approach, it is necessary to fit measurement models before testing the hypotheses by measuring convergent validity and reliability coefficients^27^. Average Variance Extracted (AVE) was calculated to measure convergent validity, indicating the correlation of a construct with its indicators. AVE critical value is 0.5^28^.

## Results

### Personal characteristics of the pastoralists

82.5% of the studied people were married and the rest were single. 37.5% of the respondents were between 45 and 55 years old with the highest frequency. The average livestock farming experience was 28.23 years, ranging between 5 and 55 years. On average, pastoralists had a 5 member family. 45% of the people were illiterate and 55% were literate enough to read and write. More than half of the pastoralists (51.2%) had between 100 and 200 heads of livestock (sheep and goats), ranging between 80 and 300 heads. 21.3% owned agricultural land and 33.8% had jobs other than animal husbandry. On average, pastoralists had an income of more than 1283 USD per year, ranging between 163 and 5000 USD per year. 73.7% of the respondents were members of rural organizations and cooperatives and 26.3% were not members of any organization (Table 4).

**Table 4:** Descriptive statistics of the personal characteristics of the pastoralists.

Characteristic	Category	Frequency	Frequency%	Min	Max	Mean
Age (year))	< 45	28	35	30	75	49.53
	45–55	30	37.5			
	55<	22	27.5			
Marital status	Married	66	82.5	-	-	-
	Single	14	17.5			
Number of household members (person)	3	17	21.3	3	9	4.94
	4	19	23.8			
	5	19	23.8			
	6	16	20			
	6<	9	11.3			
Education	Illiterate	36	45	-	-	-
	Elementary	10	12.5			
	Guidance	11	13.8			
	High school	17	21.3			
	Higher than high school	6	7.5			
Number of livestock (head)	≤ 100	24	30	80	300	152.21
	101–200	41	51.2			
	200<	15	18.8			
Animal husbandry background (year)	≤ 20	28	35	5	55	28.23
	21–30	22	27.5			
	30<	30	37.5			
Owning agricultural land	Yes	17	21.3	-	-	-
	No	63	78.8			
Having a job other than animal husbandry	Yes	27	33.8	-	-	-
	No	53	66.3			
Annual income from. animal husbandry (USD)	≤ 833	29	36.3	166	5000	1291.50
	833–1250	22	27.5			
	1250–1666	14	17.5			
	1666<	15	18.8			
Membership in rural organizations and cooperatives	Yes	59	73.7	-	-	-
	No	21	26.3			

### The performance of the natural resources participatory management and rural development project

About, 41% of the respondents (the highest frequency) evaluated the performance of the natural resources participatory management and rural development project as good. While 30, 16.3, and 12.5% of the respondents considered the performance of the project as medium, weak, and excellent, respectively (Table 5).

**Table 5:** The performance of the natural resources participatory management and rural development project.

Category	Frequency	Frequency%	Mean	SD	Min	Max
Weak (23-31.55)	13	16.3	36.91	5.36	23	48
Medium (31.56–36.91)	24	30				
Good (36.92–42.27)	33	41.3				
Excellent (42.28-48)	10	12.5				

### The relationship between personal characteristics of pastoralists and sustainable participatory management and rural development

As shown on Table 6, number of livestock (head), annual income from animal husbandry (USD), and animal husbandry background (year) had a positive and significant correlation with the implementation of sustainable management of natural resources participatory management and rural development project. In other words, with the increase of these personal characteristics, the positive outlook of pastoralists has increased on the effects of sustainable management of natural resources participatory management and rural development projects. On the other hand, educated pastoralists were less positive.

**Table 6:** Spearman’s correlation coefficients between the personal characteristics of pastoralists and sustainable management of natural resources participatory management and rural development.

Characteristic	*R*	Sig	Relationship
Age (year)	0.25*	0.017	Positive
Education	-0.10*	0.048	Negative
Number of household members (person)	-0.10	0.186	Non-significant
Number of livestock (head)	-0.33**	0.002	Positive
Animal husbandry background (year)	0.22*	0.024	Positive
Annual income from. animal husbandry (USD)	0.39**	0.000	Positive

* Significant at the 0.05, ** significant at 0.01

### The effect of participatory management project on the sustainable natural resources management

**Fig. 2:**
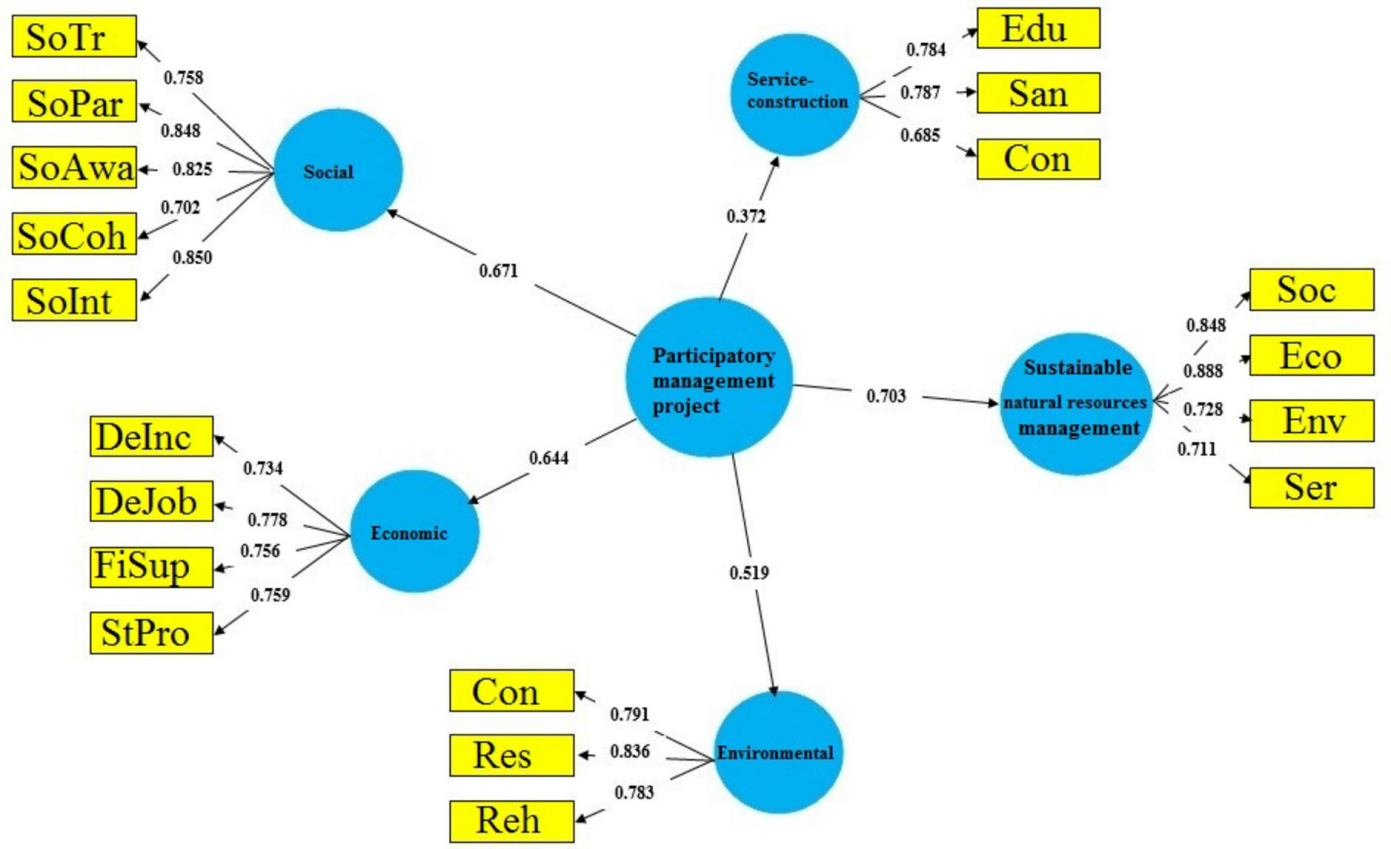
The effect of participatory management project on the sustainable natural resources management based on the path coefficient. Blue circles are latent variables. Yellow rectangles are observed variables (*SoTru* social trust,* SoPar* social participation,* SoAwa* social awareness,* SoCoh* social cohesion and solidarity,* SoInt * social interactions and communication,* DeInc* development of income sources and livelihood,* InJob* innovation in job creation and its quality,* FiSup* financial support and investment,* StPro* strengthening production and creating a market, *Con* conservation,* Res* restoration,* Reh* rehabilitation,* Edu* educational,* San* sanitary,* Con* construction- infrastructural,* Soc* social,* Eco* economic, *Env* environmental, *Ser* service-construction).

As factor load values obtained for loading indices were greater than 0.5, no indices removed. The indices “social interactions and communication” (factor load = 0.850), “innovation in job creation and its quality” (factor load = 0.778), “rehabilitation” (factor load = 0.836) “educational” (factor load = 0.787) had the greatest effect on the sustainable natural resources management (Fig. 2). The sustainable natural resources management has been more influenced by economic and social activities (Table 7).

The project has had a positive and significant impact on all criteria of sustainable natural resources management, i.e. social, Economic, environmental, and service-constructional (*P* ≤ 0.05). In other words, the implementation of the project has had the greatest impact on the economic and social criteria and the least impact on the environmental and service-constructional criteria (Table 8). The implementation of the project has had a positive and significant effect on the sustainable natural resources management (path coefficient = 0.703). In other words, the implementation of the project has explained 70.3% of the sustainable natural resources management.

**Table 7:** The factor load of observable variables based on the criteria of sustainable natural resources management.

Criterion	Cronbach’s α	CR	AVE	Indicators	Symbol	Factor load	t-value
Social	0.858	0.897	0.638	Social trust	SoTru	0.758	7.001
				Social participation	SoPar	0.848	9.850
				Social awareness	SoAwa	0.825	9.393
				Social cohesion and solidarity	SoCoh	0.702	5.092
				Social interactions and communication	SoInt	0.850	10.412
Economic	0.830	0.843	0.573	Development of income sources and livelihood	DeInc	0.734	5.674
				Innovation in job creation and its quality	InJob	0.778	9.101
				Financial support and investment	FiSup	0.756	5.782
				Strengthening production and creating a market	StPro	0.759	8.576
Environmental	0.818	0.827	0.546	Conservation	Con	0.791	5.437
				Restoration	Res	0.783	5.172
				Rehabilitation	Reh	0.836	7.695
Service-construction	0.721	0.797	0.568	Educational	Edu	0.787	4.802
				Sanitary	San	0.685	3.011
				Construction- infrastructural	Con	0.784	4.172
Sustainable natural resources management	0.808	0.873	0.635	Social	Soc	0.847	8.850
				Economic	Eco	0.888	10.736
				Environmental	Env	0.728	8.828
				Service-construction	Ser	0.711	406

**Table 8:** Path analysis showing the effect of participatory management project on sustainable natural resources management.

Path	Coefficient	t value	Sig
Social performance of participatory management project	0.671**	12.146	0.00
Economic performance of participatory management project	0.644**	8.615	0.00
Environmental performance of participatory management project	0.519**	7.184	0.00
Service-constructional performance of participatory management project	0.372**	3.512	0.00
Sustainable natural resources management performance of participatory management project	0.703**	12.756	0.00

* |t|>1.96 Significant at 0.05, ** |t|>2.58 Significant at 0.01

## Discussion

### The performance of natural resources participatory management and rural development project

As the results showed (Table 5), the performance of natural resources participatory management and rural development project was evaluated as good. Holding numerous meetings and educational and extension courses by the facilitators and extension specialists^29^, using the participation capacity of local communities, and creating new job opportunities were the most important reasons for good performance of the project. The implementation of the natural resources participatory management and rural development project had increased the level of trust and social cohesion through influencing cultural-social issues and involving people in plans and projects, so that it can take advantage of their participation potential in various activities. In other words, the strengthening of social capital through the interactions and trust of internal and external actors is one of the effects of participatory projects^30,31^. In fact, participation changes social relations and group actions, and leads to social capital improvement^17^. The continuation of the planning process has indirectly reduced the dependence of local communities, especially pastoralists, on natural resources and prevented land degradation through the boom of forgotten businesses, creating job opportunities and new sources of income in the region. Participation plays an influential role in empowering of local communities^32^, which has a positive feedback i.e., local communities’ empowerment amplifies their participation motivation^22^. The participatory management of natural resources and rural development project had the least impact on “actualizing and promoting recreational potentials”, “reducing the dependence of local communities on natural resources” and “increasing rangeland restoration practices”. Restoration practices are effective on natural resources management^29^. Despite economic activities and the prosperity of various businesses (especially in the field of medicinal plants and high-income crops such as saffron), the natural resources participatory management and rural development project has not been successful in the ecotourism sector and tourist attraction. The lack of necessary focus on the ecotourism industry, advertising and introducing tourism potentials were the reasons for the project failure in the ecotourism sector. Meanwhile, the development of tourism can change the management^33^ and provide new job opportunities for pastoralists in the rangelands^34^. On the other hand, restoration of fountains in the rangeland, distribution of abreuvoirs in rangelands, and providing subsidized forage have been created job opportunities without reducing the desire of pastoralists to animal husbandry. Based on the results, focusing on social and economic criteria has caused neglect of the environmental criterion. In such a way that the restoration and rehabilitation operations (e.g., seeding, interseeding, shrub plantation, fertilizing, etc.) were at the end in terms of the project’s performance and their feedback was not clearly defined for the local communities, especially the pastoralists. Therefore, this caused the performance of the project to be unclear in the field of rangeland restoration and rehabilitation.

### Personal characteristics of pastoralists and sustainable participatory management of natural resources and rural development

As shown in Table 6, “age” and “animal husbandry background” had positive and significant correlations with the implementation of the sustainable participatory management of natural resources and rural development project. In other words, elderly and experienced pastoralists had a more positive opinion about the effect of project implementation on the sustainable rangeland management. This can be related to the more participation of elderly and experienced pastoralists in various project activities and observing the results obtained.

Pastoralists with higher number of livestock and higher income had a more positive opinion about the effect of the implementation of the project on the sustainable rangeland management. They were more involved in the rangeland restoration and rehabilitation activities^35^. The high level of income leads to the better financial base of pastoralists, investment opportunities for the rangeland restoration and conservation, and also better knowledge and awareness on the planned projects, affecting on the development of participatory activities^36,19^. Therefore, the high level of income has a positive feedback i.e., pastoralists with higher incomes have more opportunities to participate than those with lower incomes^37^. In addition, higher income has a positive effect on the sustainable management of rangeland ecosystem^38,29^. Pastoralists with a large number of livestock usually have more participation in the project and are more informed about the effects of rangeland restoration operations due to their sensitivity in the rangeland condition and forage production. Pastoralists owning agricultural land and having a job other than animal husbandry had a more positive opinion about the effect of the project implementation on the sustainable rangeland management. It is in line with the results of Karimi et al.^36^. Owning agricultural land has made it possible for pastoralists to have more income from the economic unit related to animal husbandry in a short time and to clearly feel the effects of the implementation of the project as the results of the cultivation and processing of high-income medicinal plants such as saffron and viper’s-buglosses and educational and economic supports of facilitators and extension specialists.

Educated pastoralists had a less positive opinion about the effect of the project implementation on the sustainable rangeland management. This can be related to their lack of experience and low participation in the project implementation, as educated pastoralists are usually the younger ones. Therefore, education is effective in the participation of local communities^39,40,22^.

### The effect of sustainable participatory management of natural resources and rural development project on the sustainable natural resources management

As shown on Fig. 2, the implementation of the sustainable participatory management of natural resources and rural development project has had a positive and significant effect on the sustainable natural resources management. Its greatest impact has been related to the social criterion (Table 7). Various researches have shown that social factors have a far greater contribution compared to other variables in the participation of local communities in the sustainable natural resources management (e.g., ^41,42^). This can be related to determining the position of local communities and pastoralists in the rangeland conservation and restoration programs, principled exploitation of rangeland to conserve water and soil, holding educational and extension courses and joint meetings of government administration experts with different strata of local communities and using their opinions in collective decision-making^35,29^, providing the ground for increasing the spontaneous participation of local communities, and changing the attitude of pastoralists towards group activities. In fact, giving importance to social factors can play a significant role in attracting the participation of pastoralists in the sustainable natural resources management^41^. The project implementation increases the intragroup trust (between local communities) and intergroup trust (between local communities and government administration experts) by increasing the relationships between actors^43^. Therefore, the created trust has improved the status of interactions and the social communication network of pastoralists in the pastoral units in the area^33,44,45^. Intragroup trust and intergroup trust are influenced by the participation of local communities^18^. Resolving the pastoralists disputes and conflicts by elders, increasing the place attachment and improving livability^1,30,29^, and strengthening the spirit of solidarity and cooperation are of the other effects of the implementation of the project on the sustainable natural resources management. The project implementation has had a positive and significant effect on social awareness, which in agreement with other studies (e.g., ^22^). Holding educational and extension courses has been the basis for increasing the level of knowledge and understanding of pastoralists about rangeland management and their familiarization with alternative sources of income^33^. The establishment of rangeland management cooperatives and rural micro funds had an important role in this matter^38,24,35^.

The project implementation had had a positive and significant effect on the economic criterion of sustainable rangeland management, especially strengthening production and creating a market. The economic profitability of projects has an important effect on the participation of local communities and their continued participation in projects^39,24^. Considering the dependence of pastoralists on livestock and rangeland, attempts were made regarding the processing livestock products, their supply to local markets, and familiarizing pastoralists with job opportunities based on the region potentials (such as concentrated herding and livestock breeding, beekeeping, and exploitation of medicinal and edible plants in rangelands) with the project implementation. Therefore, creating alternative livelihood opportunities for pastoralists leads to changes in rangeland management and sustainability^33,9,37^. It can be stated that the implementation of such projects can develop the income sources of pastoralists by maintaining livestock production and their local processing and creating new business opportunities^20,29^. Therefore, combination of conservation and restoration activities with diversification of livelihood and economic opportunities increases the probability of successful implementation of projects and higher participation of local communities^30,36^. Diversification of agricultural jobs and cultivation of medicinal plants, prosperity of handicrafts and their supply in exhibitions and festivals, development of ecotourism, and processing of non-timber forest and none-forage rangeland products were of economic innovations in creating local jobs in the area. For this reason, investments in the rural businesses and rangeland restoration projects have been increased by both the governmental organizations and local communities, which led to reverse migration in some villages. In addition, the opportunities to receive loans and facilities for pastoralists have increased with the establishment of rangeland management cooperatives and rural micro funds.

The project implementation had had a positive and significant effect on the environmental criterion of sustainable rangeland management. As the environmental criterion is one of the criteria of sustainable rangeland management, the project implementation has been relatively successful on conservation, restoration and rehabilitation operations^29,46^. Fertilization of rangelands, restoration of fountains, distribution of abreuvoirs, bee keeping, aggregation of small pastoralists, and compliance with the grazing capacity as rehabilitation activities, restoration of sloping rangelands through interseeding, cultivation of fruit trees and forage plants in sloping lands, shrub plantation, increasing the seeding area in the rangeland, increasing the planting area in the rangeland, and rainwater storage operations as restoration activities, rangeland exclosure and protection of critical areas for natural reproduction, reduction of fires in rangelands, and the use of solar panels to reduce consumption of fuel plants as conservation activities have made the project implementation to have positive effects on environmental sustainability of rangelands in the conservation, restoration, and rehabilitation.

As shown in Fig. 2, the implementation of the project has had a significant impact on the service-construction criterion of sustainable rangeland management. The participatory management and rural development project has had a positive impact on educational and construction-infrastructural activities. Its impact on sanitary activities was not significant. The focus of the participatory resource management and rural development project on participatory activities in the social, economic and environmental sectors (sustainable rangeland management criteria), considering the three sides of the triangle of livestock, rangeland, and pastoralist based on predetermined goals, and lack of necessary forecast for service-construction activities are the main reasons for the obtained results.

## Conclusion

Pastoralists are facing increasing challenges in sustainable management due to the poor condition of rangelands. Sustainable rangeland management in Iran requires approaches that support pastoralists and deal with rangeland conservation with their participation, so that sustainable management strategies can be achieved. The role of local communities in various projects has not been clear, despite the implementation of various conservation, improvement, and exploitation plans in rangelands in Iran. This important issue was considered in the international participatory management of resources and rural development project, so that all activities in the economic, social and environmental criteria are carried out with the presence and participation of stakeholders with the aim of reducing the pastoralists’ dependence on rangeland and rangeland degradation. According to the results, the implementation of the participatory management of resources and rural development project increased the cooperative motivation of pastoralists in the sustainable management of rangelands and improves social cohesion and capital by increasing intragroup trust and intergroup trust and developing social networks among pastoralists in the region. Therefore, it is suggested to take the necessary benefit from the maximum participation of the local people in advancing the multi-purpose goals of the projects through proper planning and policies, sharing them in different projects, and determining the position of pastoralists in different stages of decision-making and project implementation in a win-win situation.

## Data Availability

The datasets used and/or analyzed during the current study are available from the corresponding author on reasonable request.
